# The Dietary Intake of Wheat and other Cereal Grains and Their Role in Inflammation

**DOI:** 10.3390/nu5030771

**Published:** 2013-03-12

**Authors:** Karin de Punder, Leo Pruimboom

**Affiliations:** 1 University of Girona, Plaça Sant Domènec, 3 Edifici Les Àligues, 17071 Girona, Spain; E-Mail: k.d.punder@nki.nl; 2 Uni for Life, University of Graz, Beethovenstraβe 9, 8010 Graz, Austria

**Keywords:** cereal grains, celiac disease, gluten, gliadin, inflammation, intestinal permeability, lectins, wheat, wheat germ agglutinin

## Abstract

Wheat is one of the most consumed cereal grains worldwide and makes up a substantial part of the human diet. Although government-supported dietary guidelines in Europe and the U.S.A advise individuals to eat adequate amounts of (whole) grain products per day, cereal grains contain “anti-nutrients,” such as wheat gluten and wheat lectin, that in humans can elicit dysfunction and disease. In this review we discuss evidence from *in vitro*, *in vivo* and human intervention studies that describe how the consumption of wheat, but also other cereal grains, can contribute to the manifestation of chronic inflammation and autoimmune diseases by increasing intestinal permeability and initiating a pro-inflammatory immune response.

## 1. Introduction

Inflammation is the response of the innate immune system triggered by noxious stimuli, microbial pathogens and injury. When a trigger remains, or when immune cells are continuously activated, an inflammatory response may become self-sustainable and chronic. Chronic inflammation has been associated with many medical and psychiatric disorders, including cardiovascular disease, metabolic syndrome, cancer, autoimmune diseases, schizophrenia and depression [1,2,3]. Furthermore, it is usually associated with elevated levels of pro-inflammatory cytokines and acute phase proteins, such as interferons (IFNs), interleukin (Il)-1, Il-6, tumor necrosis factor-α (TNF-α), and C-reactive protein (CRP). While clear peripheral sources for this chronic inflammation are apparent in some conditions (*i.e.*, fat production of cytokines in the metabolic syndrome), in other disorders, such as major depression, the inflammatory source is not completely understood. Genetic vulnerability, psychological stress and poor dietary patterns have all been repeatedly implicated as being of significant importance in the development of an inflammatory phenotype [3,4,5]. Dietary factors associated with inflammation include a shift towards a higher *n*-6:*n*-3 fatty acid ratio [5] and a high intake of simple sugars [6]. Other substances in our daily food, like those found in wheat and other cereal grains, are also capable of activating pro-inflammatory pathways.

## 2. Wheat Grain, Gluten and Disease

### 2.1. Wheat Allergy and Intolerance

The ingestion of wheat products has been reported to be responsible for IgE-mediated allergic reactions. Wheat-dependent exercise-induced anaphylaxis is a syndrome in which the ingestion of a product containing wheat followed by physical exercise can result in an anaphylactic response. Several proteins present in wheat, most notably gluten proteins have been shown to react with IgE in patients [7]. Other allergic responses that appear to be related to a range of wheat proteins include baker’s asthma, rhinitis and contact urticaria [7,8].

More common than wheat allergies are conditions involving wheat intolerance, including celiac disease (CD), which is estimated to affect 1% of the population of Western Europe, and dermatitis herpetiformis, which has an incidence between about 2-fold and 5-fold lower than CD [9]. The close association between type 1 diabetes and CD [10] and the observation that autoimmune diseases seem to be more prevalent in celiac patients and their relatives [11] thus links the intake of wheat with several other conditions.

### 2.2. Wheat Grain and Gluten

Gluten is the main structural protein complex of wheat consisting of glutenins and gliadins. When wheat flour is mixed with water to form dough, the gluten proteins form a continuous network which provides the cohesiveness and viscoelasticity that allows dough to be processed into bread, noodles and other foods. The protein contents of wheat varies between 7% and 22% with gluten constituting about 80% of the total protein of the seed [9]. Glutenins are the fraction of wheat proteins that are soluble in dilute acids and are polymers of individual proteins. Prolamins are the alcohol-soluble proteins of cereal grains and are specifically named gliadins in wheat. Gliadins are monomeric proteins and are classified into three groups: α/β-gliadins, γ-gliadins, and ω-gliadins [7].

### 2.3. Gluten, Gliadin and CD

Gliadin epitopes from wheat gluten and related prolamins from other gluten-containing cereal grains, including rye and barley, can trigger CD in genetically susceptible people. The symptoms of this disease are mucosal inflammation, small intestine villous atrophy, increased intestinal permeability and malabsorption of macro- and micronutrients. CD, a chronic inflammatory disorder mediated by T-cells, is preceded by changes in intestinal permeability and pro-inflammatory activity of the innate immune system. Gliadin immunomodulatory peptides can be recognized by specific T-cells, a process that can be enhanced by the deamidation of gliadin epitopes by tissue transglutaminases that convert particular glutamine residues into glutamic acid resulting in a higher affinity for HLA-DQ2 or DQ8 expressed on antigen-presenting cells (APC) [10]. Serum antibodies, among which are antibodies against tissue transglutaminases, are also found in CD. The HLA-DQ2 or HLA-DQ8 is expressed in 99.4% of the patients suffering from CD [10], however, interestingly enough, there is a group of HLA-DQ2/DQ8-negative patients suffering from gastrointestinal symptoms that respond well to a gluten-free diet. This group of “gluten-sensitive” patients does not have the CD serology and histopathology, but does present the same symptoms and shows improvements when following a gluten-free diet [12,13].

### 2.4. Gliadin and Immunity

There are at least 50 gliadin epitopes that exert immunomodulatory, cytotoxic and gut-permeating activities that can be partially traced back to different domains of α-gliadin. Where some immunomodulatory gliadin peptides activate specific T-cells, others are able to induce a pro-inflammatory innate immune response [10]. Stimulation of immune cells by gliadin is not only restricted to CD patients; the incubation of peripheral blood mononuclear cells (PBMC) from healthy HLA-DQ2-positive controls and CD patients with gliadin peptides stimulated the production of IL-23, IL-1β and TNF-α in all donors tested. Nevertheless, the production of cytokines was significantly higher in PBMC derived from CD patients [14]. Similar results were obtained by Lammers *et al.* [15], who demonstrated that gliadin induced an inflammatory immune response in both CD patients and healthy controls, though IL-6, Il-13 and IFN-γ were expressed at significantly higher levels in CD patients. IL-8 production was only expressed in a subset of healthy and CD individuals after stimulation with a specific gliadin peptide and appeared to dependent on the CXCR3 chemokine receptor only in CD patients. Sapone *et al.* [16] showed that in a subset of CD patients, but not in gluten-sensitive patients (with 36% of the studied individuals in this group being HLA-DQ2/DQ8-positive), there is an increased IL-17 mRNA expression in the small-intestinal mucosa compared to healthy controls. The same group showed that in a subset of gluten-sensitive patients (with about 50% of the studied individuals being HLA-DQ2/DQ8-positive) there is a prevailing stimulation of the innate immune system, while in CD, both the innate and adaptive immune system are involved [13].

### 2.5. Gliadin and Intestinal Permeability

In order for gliadin to interact with cells of the immune system, it has to overcome the intestinal barrier. Gliadin peptides cross the epithelial layer by transcytosis or paracellular transport. Paracellular transport occurs when intestinal permeability is increased, a feature that is characteristic for CD [17]. It is indicated by several studies that increased intestinal permeability precedes the onset of CD and is not just a consequence of chronic intestinal inflammation [18,19]. Gliadin has been demonstrated to increase permeability in human Caco-2 intestinal epithelial cells by reorganizing actin filaments and altering expression of junctional complex proteins [20]. Several studies by Fasano *et al.* show that the binding of gliadin to the chemokine receptor CXCR3 on epithelial IEC-6 and Caco2 cells releases zonulin, a protein that directly compromises the integrity of the junctional complex [21,22]. Although zonulin levels were more up-regulated in CD patients, zonulin was activated by gliadin in intestinal biopsies from both CD and non-CD patients [21,22], suggesting that gliadin can increase intestinal permeability also in non-CD patients, yet increased intestinal permeability was not observed in a group of gluten-sensitive patients [13].

## 3. Increased Intestinal Permeability

### 3.1. Increased Intestinal Permeability is Associated with Disease

Chronically increased intestinal permeability (or leaky gut syndrome) allows for the increased translocation of both microbial and dietary antigens to the periphery which can then interact with cells of the immune system. Shared amino acid motifs among exogenous peptides (HLA-derived peptides and self-tissue) may produce cross-reactivity through immunological mimicry, thereby disturbing immune tolerance in genetically susceptible individuals [23]. Not surprisingly, increased intestinal permeability has been associated with autoimmune diseases, such as type 1 diabetes [24], rheumatoid arthritis, multiple sclerosis [18], but also with diseases related to chronic inflammation like inflammatory bowel disease [18,25], asthma [26], chronic fatigue syndrome and depression. The latter two conditions see patients with significantly greater values of serum IgA and IgM to LPS of gram-negative enterobacteria compared to controls, implying intestinal permeability is increased in these patients [27,28,29].

### 3.2. Intestinal Barrier Function and Inflammation

**Figure 1:**
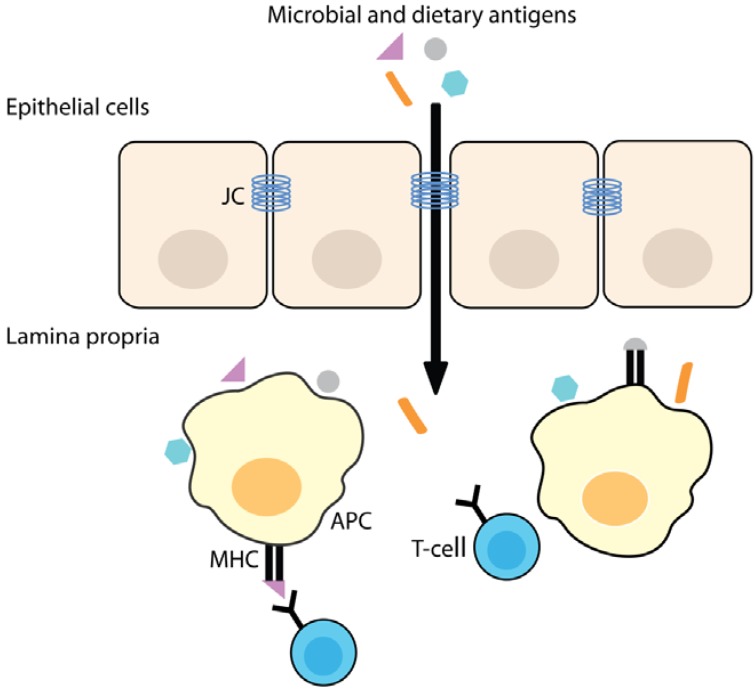
Increased intestinal permeability allows for the passage of microbial and dietary antigens across the epithelial layer into the lamina propria, where these antigens can be taken up by APC and presented to T-cells. JC, junctional complex.

The intestinal barrier allows the uptake of nutrients and protects from damage of harmful substances from the gut lumen. Macromolecules that can be immunogenic like proteins, large peptides, but also bacteria and lectins, can be endocytosed or phagocytosed by enterocytes forming the epithelial layer of the gut. Absorbed proteins will generally enter the lysosomal route and will be degraded to small peptides. Normally, only small amounts of antigen pass the barrier by transcytosis and interact with the innate and adaptive immune system situated in the lamina propria. Highly specialized epithelial microfold (M) cells function as active transporters of dietary and microbial antigens from the gut lumen to the immune system, where either a pro-inflammatory or tolerogenic immune response can be generated. The paracellular route is regulated by the junctional complex that allows the passage of water, solutes and ions, but under normal conditions provides a barrier to larger peptides and protein-sized molecules. When the barrier function is disrupted, there is an increased passage of dietary and microbial antigens interacting with cells of the immune system [25,30] (Figure 1).

### 3.3. The Role of Zonulin Signaling on Intestinal Permeability

Intestinal permeability is a measure of the barrier function of the gut which relates to the paracellular space surrounding the brush border surface of the enterocytes and the junctional complexes consisting of tight junctions, adherent junctions, desmosomes and gap junctions [31]. The junctional complexes are regulated in response to physiological and immunological stimuli, like stress, cytokines, dietary antigens and microbial products [31]. As mentioned before, zonulin, a protein identified as prehaptoglobulin-2 (the precursor of haptoglobin-2) is also a regulator of intestinal permeability. Haptoglobin-2, together with haptoglobin-1, is one of the two gene variants of the multifunctional protein haptoglobin and is associated with an increased risk for CD (homozygotes and heterozygotes) and severe malabsorption (homozygotes) [32,33]. The haptoglobulin-2/zonulin allele has a frequency of about 0.6 in Europe and the U.S.A, but varies throughout the world depending on racial origin [34].

### 3.4. High Zonulin Levels are Observed in Auto-Immune and Inflammatory Diseases

Zonulin signaling is proposed to cause rearrangements of actin filaments and induces the displacement of proteins from the junctional complex, thereby increasing permeability [18,32,35]. Gliadin peptides initiate intestinal permeability through the release of zonulin, thereby enabling paracellular translocation of gliadin and other dietary and microbial antigens, which by interacting with the immune system give rise to inflammation. In this manner, a vicious cycle is created in which, as a consequence of the persistent presence of pro-inflammatory mediators, intestinal permeability will increase even further. High zonulin levels (together with increased intestinal permeability) have been observed in autoimmune and inflammatory diseases like CD, multiple sclerosis, asthma and inflammatory bowel disease and the haptoglobin polymorphism is associated with rheumatoid arthritis, ankylosing spondylitis, schizophrenia and certain types of cancer [32].

The zonulin inhibitor Larozotide acetate was tested in an inpatient, double-blind randomized placebo-controlled trial. The group of CD patients in the placebo group that were exposed to gluten showed a 70% increase in intestinal permeability, while no changes were seen in the group exposed to Larazotide acetate. Also gastrointestinal symptoms were significantly more frequent in the placebo group [32]. These results suggest that in CD patients, when intestinal barrier function is restored, autoimmunity will disappear while the trigger (gluten) is still there. Besides gliadin from wheat gluten, the lectin wheat germ agglutinin (WGA) has also been shown to stimulate cells of the immune system and increase intestinal permeability, as we will now discuss further.

## 4. Wheat Germ Agglutinin (WGA)

### 4.1. Dietary WGA

Lectins are present in a variety of plants, especially in seeds, where they serve as defense mechanisms against other plants and fungi. Because of their ability to bind to virtually all cell types and cause damage to several organs, lectins are widely recognized as anti-nutrients within food [36]. Most lectins are resistant to heat and the effects of digestive enzymes, and are able to bind to several tissues and organs *in vitro* and *in vivo* (reviewed by Freed 1991 [37]). The administration of the lectin WGA to experimental animals caused hyperplastic and hypertrophic growth of the small intestine, hypertrophic growth of the pancreas and thymus atrophy [36]. Lectin activity has been demonstrated in wheat, rye, barley, oats, corn and rice, however the best studied of the cereal grain lectins is WGA [38].

The highest WGA concentrations are found in wheat germ (up to 0.5 g/kg [39]). Although unprocessed wheat germ, like muesli, contains far higher amounts of active WGA than do processed wheat germ products, WGA activity is still apparent in several processed breakfast cereals as assessed by hemagglutination and bacterial agglutination assays [40,41]. A summary of the amount of active WGA in commonly consumed wheat derived products is listed in Table 1.

**Table 1:** Amount of active WGA in wheat-derived products.

Wheat Derived Products	WGA μg/g (±SD)	Reference Source
Wheat germ	300 (±35)	*Vincenzi* et al., *2000* [42]
Wheat germ	100–500	*Peumans and Van Damme, 1996* [39]
Semolina ^a^	4.0 (±1.0)–10.7 (±1.5)	*Matucci* et al., *2004* [43]
Flour ^a^	4.3 (±0.7)–4.4 (±1.0)	
Wholemeal flour ^a^	29.5 (±2.5)–50 (±5.5)	
Pasta ^a^	≤0.4 (±0.2)–3.2 (±0.2)	
Pasta cooked ^a^	≤0.3 (±0.2)	
Wholemeal pasta (enriched with wheat germ)	40 (±2.7)	
Wholemeal pasta (enriched with wheat germ) cooked	Not detectable	
Wholemeal pasta ^a^	0–5.7 (±0.2)	
Wholemeal pasta cooked ^a^	Not detectable	
Breakfast cereals ^a^	13–53	*Ortega-Barria* et al., *1994* [41]

^a^ Values are obtained from more than one product and from different manufacturers.

### 4.2. WGA Binds to Cell Surface Glycoconjugates

WGA binds to *N*-glycolylneuraminic acid (Neu5Ac), the sialic acid predominantly found in humans [44], allowing it to adhere to cell surfaces like the epithelial layer of the gut. The surface of many prokaryotic and eukaryotic cells are covered with a dense coating of glycoconjugates, also named glycocalyx. Sialic acids are a wide family of nine-carbon sugars that are typically found at the terminal positions of many surface-exposed glycoconjugates and function for self recognition in the vertebrate immune system, but they can also be used as a binding target for pathogenic extrinsic receptors and molecular toxins [45,46,47]. WGA binding to Neu5Ac of the glycocalyx of human cells (and pathogens expressing Neu5Ac) allows for cell entry and could disturb immune tolerance by evoking a pro-inflammatory immune response (discussed below).

### 4.3. WGA and Immunity

WGA induces inflammatory responses by immune cells. For example, WGA has been shown to trigger histamine secretion and granule extrusion from non-stimulated rat peritoneal mast cells [48], induce NADP-oxidase activity in human neutrophils [49] and stimulate the release of the cytokines IL-4 and IL-13 from human basophils [50]. In human PBMC, WGA induced the production of IL-2, while simultaneously inhibiting the proliferation of activated lymphocytes [51]. WGA stimulated the secretion of IL-12, in a T- and B-cell-independent manner in murine spleen cells. IL-12, in turn, activated the secretion of IFN-γ by T or natural killer cells [52]. In murine peritoneal macrophages WGA induced the production of the pro-inflammatory cytokines TNF-α, IL-1β, IL-12 and IFN-γ [53]. Similar results have been observed in isolated human PBMC, given that nanomolar concentrations of WGA stimulated the release of several pro-inflammatory cytokines. In the same study a significant increase in the intracellular accumulation of IL-1β was measured in monocytes after WGA exposure [54]. These results indicate that, when delivered *in vitro*, WGA is capable of directly stimulating monocytes and macrophages, cells that have the ability to initiate and maintain inflammatory responses. Monocytic cells have been shown to engulf WGA via receptor-mediated endocytosis or by binding to non-receptor glycoproteins [55].

Human data showing the influence of WGA intake on inflammatory markers are lacking, however, antibodies to WGA have been detected in the serum of healthy individuals [56]. Significantly higher antibody levels to WGA were measured in patients with CD compared to patients with other intestinal disorders. These antibodies did not cross-react with gluten antigens and could therefore play an important role in the pathogenesis of this disease [57].

### 4.4. WGA and Intestinal Permeability

After ingestion, WGA is capable of crossing the intestinal barrier. In animal models, WGA has been shown to reach the basolateral membrane and walls of the small blood vessels in the subepithelium of the small intestine [36]. WGA can be phagocytosed by binding to membrane non-receptor glycoproteins, a process that has been observed in Caco-2 cells [58]. WGA can also be endocytosed by antigen sampling M-cells [59,60] or by enterocytes via binding to epidermal growth factor receptors [61]. Another possible route for lectin entry into the periphery is by paracellular transport, a process that can be further aggravated by the binding of gliadin to the chemokine receptor CXR3 on enterocytes.

WGA itself has been found to affect enterocyte permeability. Investigations by Dalla Pellegrina *et al.* [54] showed, *in vitro*, that exposure to micromolar concentrations of WGA impairs the integrity of the intestinal epithelial layer, allowing passage of small molecules, like lectins. At the basolateral side of the epithelium, WGA concentrations in the nanomolar range induced the secretion of pro-inflammatory cytokines by immune cells [54]. This may further affect the integrity of the epithelial layer, heightening the potential for a positive feedback loop between WGA, epithelial cells and immune cells. When combined, these mechanisms are likely able to significantly increase the percentage of consumed WGA that can cross the epithelial layer compared to the low percentage of WGA crossing by means of transcytosis (0.1%) alone [54]. This suggests that, together with gliadin, WGA can increase intestinal permeability, resulting in an increase of translocating microbial and dietary antigens interacting with cells of the immune system. 

## 5. Animal Data on Cereal Grain Intake

There are two rodent models of spontaneous type 1 diabetes: the non-obese-diabetic (NOD) mouse and the diabetes-prone BioBreeding (BBdp) rat. In these animals, a cereal-based diet containing wheat induced the development of type 1 diabetes, while animals fed a hypoallergenic diet (gluten free) or a hypoallergenic diet supplemented with casein showed a decreased incidence and a delayed onset of this disease. BBdp rats fed a cereal-based diet showed increased intestinal permeability and a significant increase in the percentage of IFN-γ-producing Th1 lymphocytes in the mesenteric lymph nodes in the gut [30]. Compared to animals fed a hypoallergenic diet, NOD mice fed a wheat-based diet expressed higher mRNA levels of the pro-inflammatory cytokines IFN-γ and TNF-α and the inflammatory marker inducible NO synthase in the small intestine. While these diet-induced changes in gut-wall inflammatory activity did not translate to increased cytokine mRNA in Peyers patches, structures that contribute to immune regulation to exogenous antigens, it is possible that the gut-signal may promote systemic inflammation via other mechanisms, such as activating intraepithelial lymphocytes and mesenteric lymph node cells [62]. These *in vivo* results show that, in two rodent models of spontaneous type 1 diabetes, a cereal-containing diet induces the (early) onset of disease and increases markers of inflammation. In addition, Chignola *et al.* [63] have shown in rats that a WGA-depleted diet was associated with reduced responsiveness of lymphocytes from primary and secondary lymphoid organs after *in vitro* stimulation and attenuated spontaneous proliferation when compared to lymphocytes from rats fed a WGA-containing diet, indicating the stimulatory effect of WGA on cells of the immune system.

## 6. Human Studies on Cereal Grain Intake and Inflammation

### 6.1. Human Epidemiological Data on Cereal Grain Intake and Inflammation

Observational prospective and cross-sectional studies show that the intake of whole grain products is associated with reduced risks for developing type 2 diabetes, cardiovascular diseases, obesity and some types of cancer [64]. Inflammation is associated with these conditions and some studies have shown that associations between the intake of whole grains and decreased inflammatory markers (CRP, Il-6) are found [65]. Intervention studies, however, do not demonstrate a clear effect of the intake of whole grains on inflammation [66,67,68,69,70,71] and it could therefore be that other components in the diet modulate the immune response.

It has been shown that the intake of whole grains is associated with healthier dietary factors and a healthier lifestyle in general. In a Scandinavian cross-sectional study, the intake of whole grains was directly associated with the length of education, the intake of vegetables, fruits, dairy products, fish, shellfish, coffee, tea and margarine and inversely associated with smoking, BMI and the intake of red meat, white bread, alcohol, cakes and biscuits [72]. Good quality epidemiological studies attempt to control these confounding factors, but with the consequence that associations are attenuated or become insignificant.

### 6.2. Human Intervention Trials on Cereal Grain Intake and Inflammation

To accurately estimate the causal relationship of cereal grain intake and inflammation, intervention trials provide us with better evidence. Wolever *et al.* [71] showed that a diet with a low glycemic index (containing whole grains) compared to high (containing refined grain products), resulted in sustained reductions in postprandial glucose and CRP levels on the long-term in patients with type 2 diabetes treated with diet alone. A refined grain is a whole grain that has been stripped of its outer shell (fiber) and its germ, leaving only the endosperm, resulting in lower levels of macro- and micronutrients and a higher dietary glycemic index for refined grains compared to whole grains. Refined wheat products contain less WGA, but still contain a substantial amount of gluten. It should be noted that whole grains contain phytochemicals, like polyphenols, that can exert anti-inflammatory effects which could possibly offset any potentially pro-inflammatory effects of gluten and lectins [73].

The substitution of whole grain (mainly based on milled wheat) for refined grains products in the daily diet of healthy moderately overweight adults for six weeks did not affect insulin sensitivity or markers of lipid peroxidation and inflammation [66]. Consistent with these finding are the results of Brownlee *et al.* [67], who showed that infrequent whole-grain consumers, when increasing whole grain consumption (including whole wheat products), responded with no improvements of the studied biomarkers of cardiovascular health, including insulin sensitivity, plasma lipid profile and markers of inflammation. The substitution of refined cereal grains and white bread with three portions of whole wheat food or one portion of whole wheat food combined with two servings of oats significantly decreased the systolic blood pressure and pulse pressure in middle-aged, healthy, overweight men and women, yet none of the interventions significantly affected systemic markers of inflammation [70]. In obese adults suffering from metabolic syndrome, there were significantly greater decreases in CRP and the percentage of body fat in the abdominal region in participants consuming whole grains compared to those consuming refined grains. It must be noted that both diets were hypocaloric (reduced by 500 kcal/d) [69]. Most of the intervention studies mentioned above attempted to increase whole grain intake and were using refined grain diets as controls, thereby making it very difficult to draw any conclusions on the independent role of cereal grains in disease and inflammation.

### 6.3. Health Effects of the Paleolithic Diet

There are few studies that investigate the influence of a paleolithic type diet comprising lean meat, fruits, vegetables and nuts, and excluding food types, such as dairy, legumes and cereal grains, on health. In domestic pigs, the paleolithic diet conferred higher insulin sensitivity, lower CRP and lower blood pressure when compared to a cereal-based diet [74]. In healthy sedentary humans, the short-term consumption of a paleolithic type diet improved blood pressure and glucose tolerance, decreased insulin secretion, increased insulin sensitivity and improved lipid profiles [75]. Glucose tolerance also improved in patients suffering from a combination of ischemic heart disease and either glucose intolerance or type 2 diabetes and who had been advised to follow a paleolithic diet. Control subjects who were advised to follow a Mediterranean-like diet based on whole grains, low-fat dairy products, fish, fruits and vegetables did not significantly improve their glucose tolerance despite decreases in weight and waist circumference [76]. Similar positive results on glycemic control were obtained in diabetic patients when the paleolithic diet was compared with the diabetes diet. Participants were on each diet for three months, whereby the paleolithic diet resulted in a lower BMI, weight and waist circumference, higher mean HDL, lower mean levels of hemoglobin A1c, triacylglycerol and diastolic blood pressure, though levels of CRP were not significantly different [77]. Although the paleolithic diet studies are small, these results suggest that, together with other dietary changes, the withdrawal of cereal grains from the diet has a positive effect on health. Nevertheless, because these studies are confounded by the presence or absence of other dietary substances and by differences in energy and macronutrient intake, factors that could all affect markers of inflammation, it is difficult to make a concise statement on the impact of cereal grains on these health outcomes.

### 6.4. Rechallenge Trial of Effects of Dietary Gluten

One human intervention study specifically focused on the effects of dietary gluten on inflammation. Biesiekierski *et al.* [12] undertook a double-blind randomized, placebo-controlled rechallenge trial to investigate the influence of gluten in individuals with irritable bowel syndrome but without clinical features of CD, who reached satisfactory levels of symptom control with a gluten-free diet. After screening the participants, about 50% of the individuals in both the gluten and placebo group were HLA-DQ2 and/or HLA-DQ8 positive. Participants received either gluten or placebo together with a gluten-free diet for six weeks. Endpoints in the study were symptom assessments and biomarkers of inflammation and intestinal permeability. The patients receiving gluten reported significantly more symptoms compared to the placebo group. The most striking outcome of this study was that for all the endpoints measured, there were no differences in individuals with or without HLA-DQ2/DQ8, indicating that the intake of gluten can cause symptoms also in individuals without this specific HLA-profile. No differences in biomarkers for inflammation and intestinal permeability were found between both groups, however, inflammatory mediators have been implicated in the development of symptoms in patients with irritable bowel syndrome [78]. It could therefore be inferred that the markers used to measure inflammation and intestinal permeability were not sensitive enough to detect subtle changes on the tissue level.

## 7. Conclusion

In the present review, we describe how the daily consumption of wheat products and other related cereal grains could contribute to the manifestation of chronic inflammation and autoimmune diseases. Both *in vitro* and *in vivo* studies demonstrate that gliadin and WGA can both increase intestinal permeability and activate the immune system. The effects of gliadin on intestinal permeability and the immune system have also been confirmed in humans. Other cereal grains containing related prolamins and lectins have not been so extensively studied and, therefore, more research investigating their impact on intestinal permeability and inflammation is required. It would be interesting to further elucidate the role of other prolamins on zonulin release and intestinal permeability.

In CD and gluten-sensitive individuals, adverse reactions to the intake of wheat, rye and barley are clinically apparent; however, it is important to gain better insights on the effects of the consumption of these cereal grains in other groups of patients and in healthy individuals. It would be of high interest to investigate the effects of the withdrawal of cereal grain products from the diet on inflammatory markers and intestinal permeability in healthy subjects and patients suffering from inflammation-related diseases and measure the same parameters in a rechallenge trial. Ideally, in such an intervention study, the diet must be completely controlled and combined with the appropriate substitution of foods in the cereal grain-deprived diet so that small dietary variations and alterations in energy intake can be avoided and cannot potentially influence inflammatory markers.

Until now, human epidemiological and intervention studies investigating the health effects of whole grain intake were confounded by other dietary and lifestyle factors and, therefore, well-designed intervention studies investigating the effects of cereal grains and their individual components on intestinal permeability and inflammation are warranted.
